# CD4/CD8 ratio is not predictive of multi-morbidity prevalence in HIV-infected patients but identify patients with higher CVD risk

**DOI:** 10.7448/IAS.17.4.19709

**Published:** 2014-11-02

**Authors:** Marianna Menozzi, Stefano Zona, Antonella Santoro, Federica Carli, Chiara Stentarelli, Cristina Mussini, Giovanni Guaraldi

**Affiliations:** Infectious Disease Clinic, AOU Policlinico di Modena, Modena, Italy

## Abstract

**Background:**

CD4/CD8<0.8 is a surrogate marker of immune-activation/immunosenescence and independently predicts mortality in the HIV-infected patients due to non-AIDS related events. Most studies showed that patients on antiretroviral therapy (ART) often fail to normalize the CD4/CD8 ratio despite CD4 count normalization. Primary objective of the study was to explore the impact of CD4/CD8<0.8 as independent predictor of HIV-associated non-AIDS (HANA) conditions and multimorbidity (MM) in HIV patients. In patients with no previous history of cardiovascular disease (CVD) a particular insight is provided in the association between impact of CD4/CD8<0.8 and risk prediction of CVD or radiological markers of subclinical CVD.

**Materials and Methods:**

914 consecutive patients attending Modena Metabolic HIV Clinic were evaluated in a cross-sectional retrospective study. Inclusion criteria: stable ART from ≥2 years; HIV-RNA plasma levels<40 copies/mL; stable CD4 count≥350/mmc. CD4/CD8 strata (0.8) was chosen as a cut off representing the median value of the cohort. MM was defined as the presence of≥2 HANA conditions including standard defined: chronic kidney disease, hypertension, previous CVD events, osteoporosis and diabetes mellitus. Calendar year of ART initiation was defined: “PreART” (<2000); “EarlyART” (2000–2005) and “LateART” (>=2006). High CVD risk was defined for Framingham Risk Score (FRS)≥6. Subclinical CVD was defined using cardiac CT scan for calcium score (CAC)≥100. Logistic univariate and multivariable adjusted analysis were performed to assess relationships between variables.

**Results:**

Demographic and HIV-specific variables distribution in patients with and without MM are shown in Table 1.

Figure 1 shows HANA distribution across CD4/CD8 strata: CVD prevalence only appeared to be higher in patients with no CD4/CD8>0.8.In multivariable analyses CD4/CD8<0.8 was not an independent predictor of MM (OR=1.225, CI 0.891; 1.681, p=0.211) after adjustment for age, gender and BMI. Patients with CD4/CD8<0.8 displayed higher CVD risk but not higher prevalence of subclinical CVD. At multivariable analyses CD4/CD8<0.8 remained predictor of higher CVD risk (OR=0.65, CI 0.47–0.917, p=0.014) after correction for sex, BMI, age strata and HIV infection duration.

**Conclusions:**

Low CD4/CD8 ratio was not associated with MM prevalence. Patients with CD4/CD8<0.8 ratio displayed higher prevalence of CVD. At multivariable logistic regression CD4/CD8<0.8 is an independent prepredictor of enhanced CVD risk. This may support role of immune-activation/senescence in the pathogenesis of CVD.

**Table 1:** Patients demographic, anthropometric and HIV specific characteristics

	MM−	MM+	P value
Total Number	665 (72.5%)	249 (27.5%)	–
Women	200 (30.1%)	48 (19.3%)	0.001
Age	47 (44-51)	52 (48-58)	<0.001
CDC C stage	147 (23.5%)	72 (29.5%)	0.037
Smoking status			0.001
Non-smoker	376 (57.5%)	171 (69%)	
<10 cigarettes/day	105 (16.1%)	40 (16.1%)	
>10 cigarettes/day	173 (26.5%)	37 (14.9%)	
Sedentary life	376 (57.5%)	171 (69%)	0.351
No alcohol intake	349 (53.4%)	160 (34.5%)	0.002
BMI	23 (21–25)	24 (21-26)	0.010
Fasting glucose	91 (86–98)	99 (89–114	<0.001
Triglycerides	139 (98–199)	164 (111–220)	0.028
Total Cholesterol	199 (171–227)	196 (169–222)	0.481
HDL Cholesterol	47 (38–57)	45 (36.53)	0.014
HIV duration (months)	213 (149–300)	217 (174–273)	0.017
CD4 nadir	210 (104–300)	172 71-284)	0.022
Current CD4	652 (508–814)	628 (483–803)	0.538
Current CD8	867 (650–1127)	890 (629–1200)	0.068
Current CD4/CD8 ratio	0.76 (0.57–1.03)	0.76 (0.51–1.09)	0.489
CD4/CD8 ratio strata			0.373
<0.8	373 (56.1%)	136 (54.6%)	
>=0.8	292 (43.9%)	113 (45.5%)	
ARV initiation			0.049
PreART	383 (58.1%)	157 (63.1%)	
EarlyART	168 (25.5%)	67 (26.9%)	
LateART	108 (16.4%)	25 (10%)	
Age at ARV initiation	36 (+-7)	41 (+-9)	0.001

**Figure 1:**
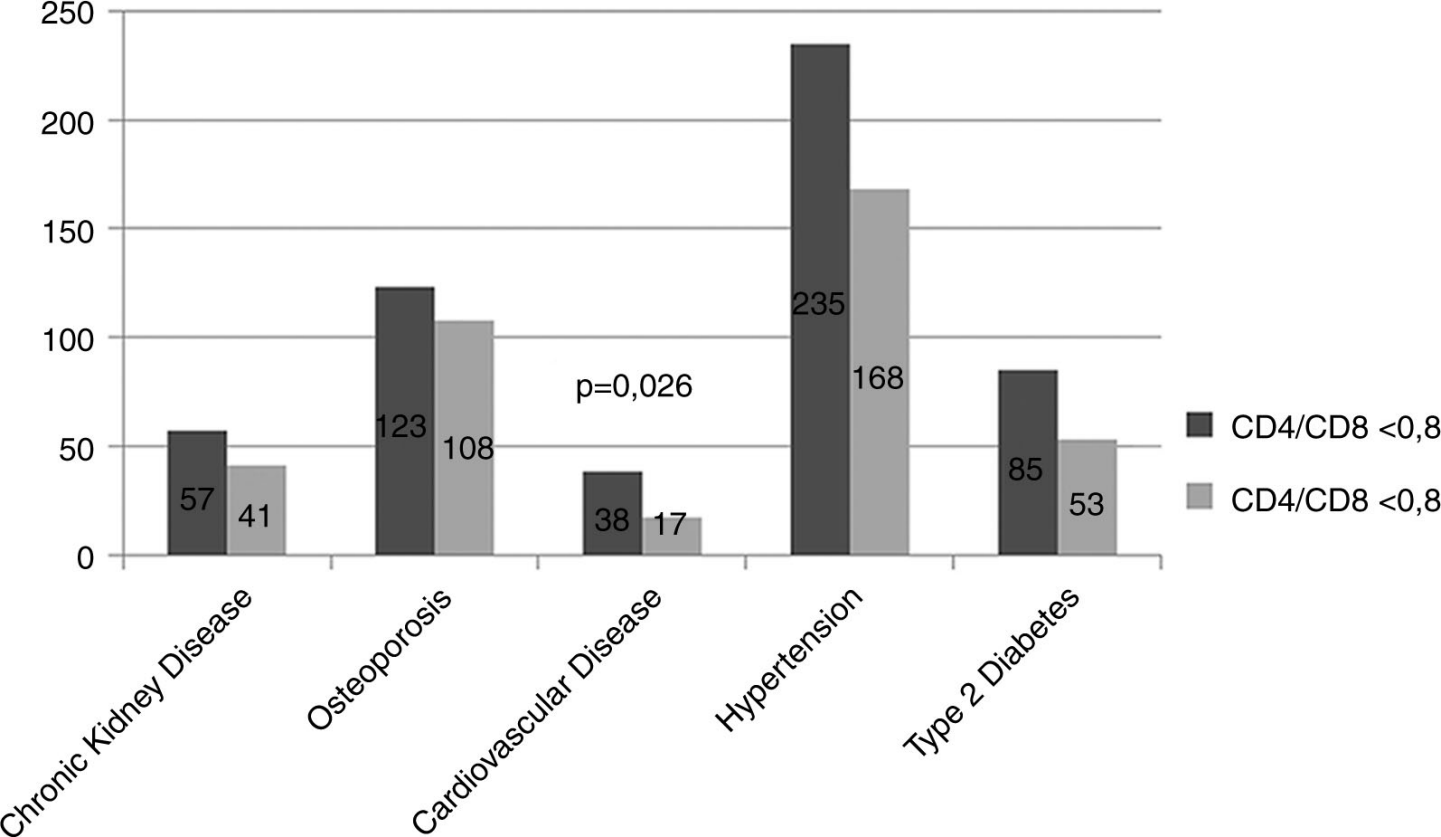
HANA distribution across CD4/CD8 strata.

